# Supplementary material for: An ELOVL2-Based Epigenetic Clock for Forensic Age Prediction: A Systematic Review
Source: Int J Mol Sci. 2023 Jan 23;24(3):2254. doi: 10.3390/ijms24032254 (PMC9916975; doi:10.3390/ijms24032254)
Supplement: Supplementary file 1 [file ijms-24-02254-s001.zip › Paparazzo et al revised/ijms-2127076-supplementary.pdf]

International Journal of
Molecular Sciences

Supplementary Materials

Supplementary Figures

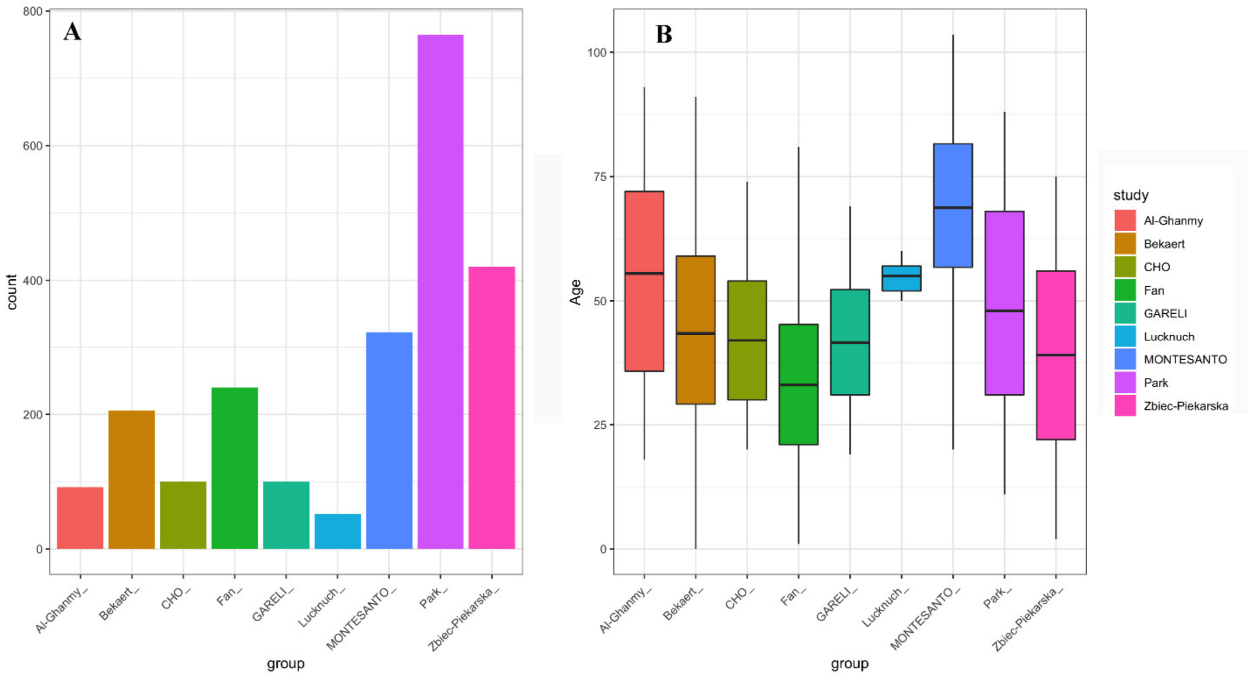

Supplementary Figure S1. (**A**) number of samples collected in the 9 different datasets and (**B**) corresponding age distribution.

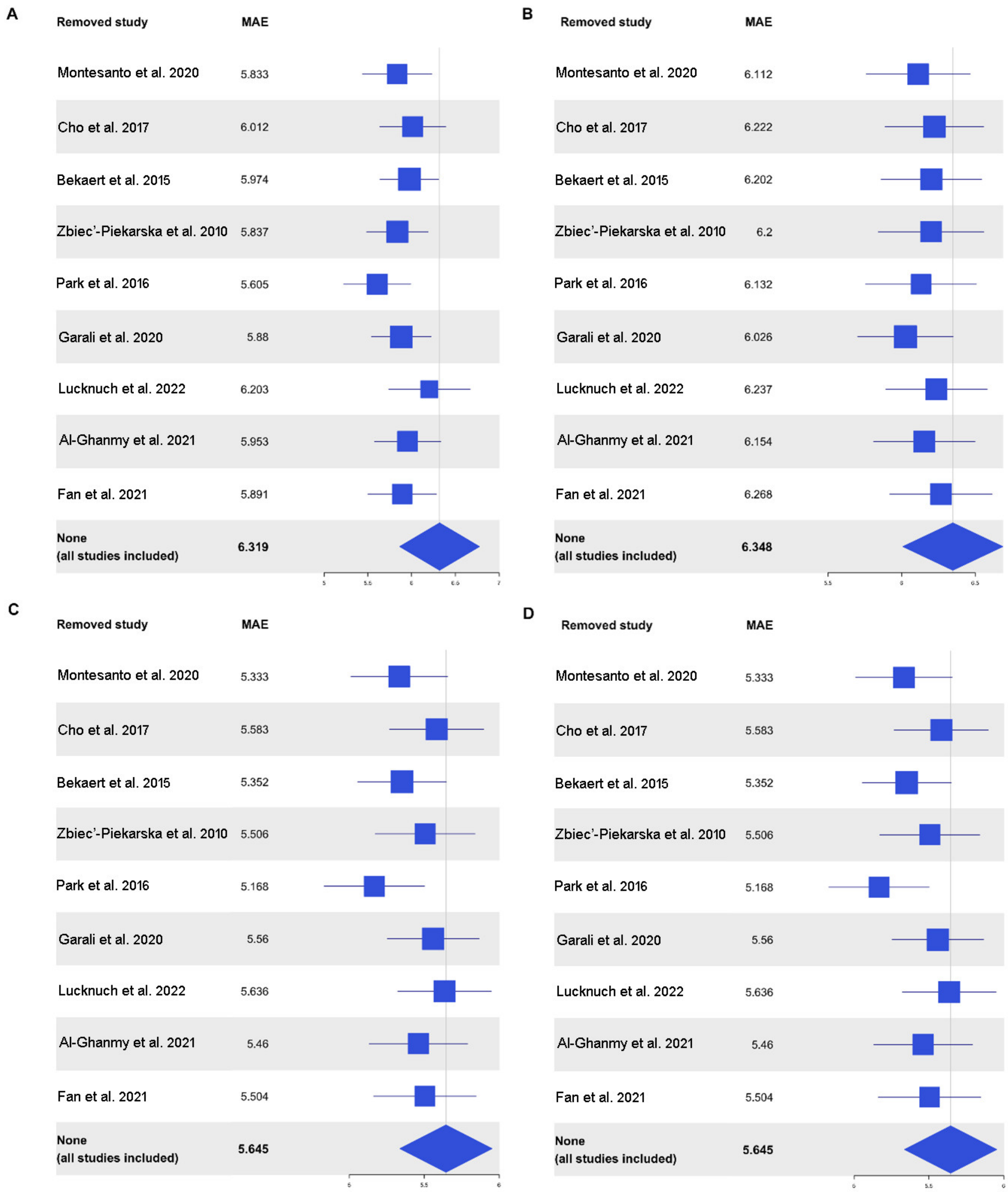

Supplementary Figure S2. Forest plots presenting the results of the sensitivity analysis for the Multiple Quadratic Regression (A); Principal Components (B); Support Vector Machine with radial kernel (C); Gradient Boosting Regression (D). Each row reports the results obtained removing a single dataset, expect for the final row which presents the results on the whole sample. Results are reported as mean absolute error (MAE), along with a graphical representation of the MAE and its confidence interval.

Supplementary Tables

Supplementary Table S1. classification performances of the tested models in the German (A) and in the Japanese populations (B). The elements on the principal diagonal represent the MAE values of the corresponding model. The content in the upper triangle is represented by the p values from the t-test comparing the two corresponding models. The content in the lower triangle is represented by the difference (95% CI in parenthesis) between the performances of the two compared models.

A)

	MLR	MQR	SVM	GBR	PC
MLR	22.714	0	0	0	3.00E-04
MQR	1.857 (1.208, 2.515)	20.857	0	0	0.0734
SVM	4.32 (3.333, 5.337)	2.463 (1.588, 3.334)	18.394	0.0663	0
GBR	4.876 (3.947, 5.81)	3.019 (2.316, 3.711)	0.556 (-0.038, 1.162)	17.838	0
PC	1.277 (0.56, 2.016)	-0.58 (-1.225, 0.067)	-3.043 (-3.897, -2.201)	-3.599 (-4.312, -2.89)	21.437

B)

	MLR	MQR	SVM	GBR	PC
MLR	25.328	0.1772	0	0	0.624
MQR	1.198 (-0.505, 2.937)	24.13	0	0	0.2543
SVM	5.081 (2.876, 7.549)	3.883 (2.259, 5.619)	20.248	0.5133	0
GBR	5.531 (3.798, 7.354)	4.333 (3.031, 5.697)	0.45 (-0.922, 1.77)	19.797	0
PC	0.452 (-1.025, 2.256)	-0.746 (-1.954, 0.581)	-4.629 (-6.113, -3.151)	-5.079 (-6.402, -3.725)	24.876

Supplementary Methods

Derivation of the best models for comparison against Garali et al.

Each study was held out in turn for performance estimation while the remaining data were split between a training and a validation set (50% of samples in each split). Linear model, multiple quadratic model, PC model, SVM and GBR were then fitted on the training set and the best algorithm was identified by applying these models on the validation set. The optimal models for comparison against Garali et al. [16] were fitted by applying the best algorithm on training and validation set combined. Notably, the holdout sets were never used for deriving any of the models.
